# Pyruvate dehydrogenase complex deficiency: updating the clinical, metabolic and mutational landscapes in a cohort of Portuguese patients

**DOI:** 10.1186/s13023-020-01586-3

**Published:** 2020-10-22

**Authors:** Hana Pavlu-Pereira, Maria João Silva, Cristina Florindo, Sílvia Sequeira, Ana Cristina Ferreira, Sofia Duarte, Ana Luísa Rodrigues, Patrícia Janeiro, Anabela Oliveira, Daniel Gomes, Anabela Bandeira, Esmeralda Martins, Roseli Gomes, Sérgia Soares, Isabel Tavares de Almeida, João B. Vicente, Isabel Rivera

**Affiliations:** 1 Metabolism and Genetics Group, Research Institute for Medicines (iMed.ULisboa), Faculty of Pharmacy, Universidade de Lisboa, Av. Prof. Gama Pinto, 1649-003 Lisboa, Portugal; 2 Department of Biochemistry and Human Biology, Faculty of Pharmacy, Universidade de Lisboa, Lisboa, Portugal; 3 Department of Pediatrics, Hospital D. Estefânia, Lisbon, Portugal; 4 Department of Pediatrics, Hospital Santa Maria, Lisbon, Portugal; 5 Department of Medicine, Hospital Santa Maria, Lisbon, Portugal; 6 Department of Pediatrics, Hospital Santo António, Porto, Portugal; 7 Department of Neuropediatrics, Hospital Pedro Hispano, Matosinhos, Portugal; 8 Instituto de Tecnologia Química e Biológica António Xavier, NOVA University of Lisbon, Av. da República (Estação Agronómica Nacional), 2780-157 Oeiras, Portugal

**Keywords:** Pyruvate dehydrogenase complex deficiency, Neurological dysfunction, Lactic acidosis, Mutational analysis, Genotype–phenotype correlation

## Abstract

**Background:**

The pyruvate dehydrogenase complex (PDC) catalyzes the irreversible decarboxylation of pyruvate into acetyl-CoA. PDC deficiency can be caused by alterations in any of the genes encoding its several subunits. The resulting phenotype, though very heterogeneous, mainly affects the central nervous system. The aim of this study is to describe and discuss the clinical, biochemical and genotypic information from thirteen PDC deficient patients, thus seeking to establish possible genotype–phenotype correlations.

**Results:**

The mutational spectrum showed that seven patients carry mutations in the *PDHA1* gene encoding the E1α subunit, five patients carry mutations in the *PDHX* gene encoding the E3 binding protein, and the remaining patient carries mutations in the *DLD* gene encoding the E3 subunit. These data corroborate earlier reports describing *PDHA1* mutations as the predominant cause of PDC deficiency but also reveal a notable prevalence of *PDHX* mutations among Portuguese patients, most of them carrying what seems to be a private mutation (p.R284X). The biochemical analyses revealed high lactate and pyruvate plasma levels whereas the lactate/pyruvate ratio was below 16; enzymatic activities, when compared to control values, indicated to be independent from the genotype and ranged from 8.5% to 30%, the latter being considered a cut-off value for primary PDC deficiency. Concerning the clinical features, all patients displayed psychomotor retardation/developmental delay, the severity of which seems to correlate with the type and localization of the mutation carried by the patient. The therapeutic options essentially include the administration of a ketogenic diet and supplementation with thiamine, although arginine aspartate intake revealed to be beneficial in some patients. Moreover, in silico analysis of the missense mutations present in this PDC deficient population allowed to envisage the molecular mechanism underlying these pathogenic variants.

**Conclusion:**

The identification of the disease-causing mutations, together with the functional and structural characterization of the mutant protein variants, allow to obtain an insight on the severity of the clinical phenotype and the selection of the most appropriate therapy.

## Background

Pyruvate dehydrogenase complex (PDC) deficiency, first described in 1970 by Blass et al. [1], is an inborn error of mitochondrial energy metabolism. The pyruvate oxidation route, that bridges the cytosolic glycolytic pathway and the mitochondrial tricarboxylic acid cycle [2], involves not only PDC but also pyruvate transport and the ancillary metabolic routes associated with various cofactors. PDC, which irreversibly decarboxylates pyruvate to acetyl coenzyme A, comprises three functional (E1 or pyruvate dehydrogenase, E2 or dihydrolipoamide transacetylase, and E3 or dihydrolipoyl dehydrogenase) and one structural (E3BP or E3 binding protein, formerly designated by protein X) components, and the regulatory PDC kinases and PDC phosphatases, which control the complex activity via phosphorylation/dephosphorylation [3]. The E1 subunit catalyzes the first, rate-limiting and irreversible step of the reaction which takes place in two active sites formed at the interface between their two α and two β subunits, each requiring a thiamine pyrophosphate cofactor and magnesium ions for activity [4, 5]^.^

The great majority (≈ 77%) of patients with PDC deficiency harbor mutations in the X-linked *PDHA1* gene which encodes the E1α subunit. Hemizygous males are usually symptomatic whereas heterozygous females present variable expression due to random patterns of X-inactivation in different tissues, the clinical manifestations depending on the proportion of cells expressing the mutant E1α subunit. The remaining cases are caused by mutations in the genes encoding the remaining subunits: *PDHB* encoding E1β (4.3%), *DLAT* encoding E2 (1.5%), *DLD* encoding E3 (6.2%) and *PDHX* encoding E3BP (10.7%) [6]^.^

The clinical presentation is extremely heterogeneous, ranging from a fatal lactic acidosis and progressive neurological and neuromuscular degradation in the neonatal period to a chronic neurological dysfunction and neurodegenerative condition. Inadequate removal of lactate but mainly of pyruvate [7] results in lactic acidemia which, together with the blood lactate/pyruvate ratio ≤ 20, is recognized as an important biomarker [8, 9]. The primary phenotypic manifestation corresponds to an impairment of neurological and/or developmental functions, leading to a wide range of symptoms and clinical features: hypotonia, seizures, ataxia, respiratory distress (like apnea and hypoventilation), facial dysmorphism, and peripheral neuropathy. Structural and functional brain abnormalities are also described, including ventriculomegaly and Leigh syndrome. According to their severity, the phenotypes can be divided into three main categories: neonatal, infantile and benign [10–12].

The therapeutic strategies target: (i) the metabolic pathway, bypassing energy production via a ketogenic diet [11, 13], (ii) the regulatory system of the dysfunctional PDC, using xenobiotic inhibitors [14, 15], or (iii) stimulation of the residual PDC activity with supplementation of cofactors [16, 17]. Nevertheless, none of these treatments is sufficiently effective and the responsiveness is markedly individual. Thus, the precise diagnosis, including the identification of the genetic defect and the phenotypic consequences, is becoming increasingly decisive for selecting the appropriate therapeutic strategy. Knowledge on the functional and structural impact of the identified mutations on the resulting PDC component variant provides relevant information for informed decisions on the therapeutic approaches.

In this study, we describe and discuss the profiles of thirteen Portuguese PDC deficient patients with reference to their clinical data, biochemical findings and results of DNA analysis, thus seeking to establish possible genotype–phenotype correlations. This is the first report on phenotypic and genotypic variability in a subset of PDC deficient patients diagnosed in Portugal.

## Results

### General information

The broad phenotypic spectrum of PDC deficiency, common to many other genetic mitochondrial disorders, often hampers the achievement of a rapid diagnosis. Patients were suspected to be PDC deficient based on clinical signs /symptoms and biochemical data, namely elevated plasma lactate (L) and pyruvate (P) levels with low L/P ratio and/or impaired PDC activity, but only those with genetic confirmation were considered in this study. Thus, a total of 13 individuals, all of European ancestry, were diagnosed (Table 1). The prevalence of PDC deficiency in Portugal is therefore estimated to be 1/790,000, comparable to the prevalence of 1/1,000,000 reported by Orphanet, the portal of rare diseases and orphan drugs, and also to the values calculated for the French population (1/827,000 – 1/1,135,000) [8, 18].

**Table 1:** Genetic, biochemical, clinical and therapeutics data concerning the cohort of 13 Portuguese PDC deficient patients

Patient	1-JCF	2-MJG	3-DMN	4-GCN^a^	5-RCN^a^	6-JAR	7-ARV	8-GMR	9-MJP	10-LCM	11-AVB	12-MBS	13-MMS
*General information*													
Year of birth	1996	2000	2005	2011	2011	2015	1983	2002	2018	2015	1988	2004	2001
Gender	M	M	M	M	M	M	F	M	F	F	F	F	F
Consanguinity	N	N	NA	N	N	N	N	Y	Y	N	N	Y	N
Age of onset (years)	1.9	2	Neonatal period	0.6	Neonatal period	0.5	1	0.5	Neonatal period	0.5	Neonatal period	Neonatal period	0.4
Age of diagnosis (years)	9	5.3	0.4	0.6	0.6	1	6	2	Neonatal period	3.3	Neonatal period	0.5	17
Actual age (years)	23	19	14	8	8	Deceased at 3.3	36	17	1	4	31	15	18
*Genetic findings*													
Affected gene	*PDHA1*	*PDHA1*	*PDHA1*	*PDHA1*	*PDHA1*	*PDHA1*	*PDHA1*	*PDHX*	*PDHX*	*PDHX*	*PDHX*	*PDHX*	*DLD*
Affected protein componente	E1α	E1α	E1α	E1α	E1α	E1α	E1α	E3BP	E3BP	E3BP	E3BP	E3BP	E3
Nucleotide exchange	c.615C>G	c.757A>G	c.905G>A	c.1132C>T	c.1132C>T	c.1132C>T	c.1133G>A	c.850C>T/c.850C>T	c.850C>T/c.850C>T	c.850C>T/c.850C>T	c.850C>T/c.483delC	c.160+1G>A/c.160+1G>A	c.259C>T/c.803_804delAG
Precursor protein Exchange	p.F205L	p.R253G	p.R302H	p.R378C	p.R378C	p.R378C	p.R378H	p.R284X/p.R284X	p.R284X/p.R284X	p.R284X/p.R284X	p.R284X/p.P161Pfs*17	p.G26Vfs*7/p.G26Vfs*7	p.P87S/p.Q268Rfs*3
Localization of mutation	Exon 7	Exon 7	Exon 10	Exon 11	Exon 11	Exon 11	Exon 11	Exon 7	Exon 7	Exon 7	Exon 7/Exon 4	Intron 1	Exon 4/Exon 9
Mutation type	Missense	Missense	Missense	Missense	Missense	Missense	Missense	Nonsense	Nonsense	Nonsense	Nonsense/frameshift	Splicing	Missense/frameshift
*Biochemical findings*													
Highest plasma lactate (mmol L^−1^)	7.9	4.0	9.1	9.4	6.6	8.9	4.5	3.5	17.0	3.0	4.4	12.2	3.0
Highest plasma pyruvate (mmol L^−1^)	0.527	0.300	0.398	NA	0.810	0.631	0.274	0.331	0.610	0.270	0.270	0.569	NA
Ratio L/P in same sample	14	12	20	NA	8	14	16	16	14	9	16	16	NA
Enzyme activity in lymphocytes	30	13	25^b^	28	40	8.5	19	30	25	30	21.3/16.3^b^	29	29
*Clinical features*													
DD/MR/PMR	Moderate	Mild	Severe	Severe	Severe	Moderate	Moderate	Severe	Severe	Severe	Severe	Severe	Moderate
Hypotonia	Y	Y	Y	Y	Y	Y	Y	Y	Y	Y	N	Y	Y
Seizures	N	N	Y	Y	Y	Y	Y	Y	N	N	N	N	Y
Microcephaly	Y	N	Y	N	N	N	Y	N	Y	Y	N	N	N
Dystonia	Y	N	Y	Y	Y	Y	N	Y	N	N	Y	Y	Y
Ataxia	Y	Y	N	Y	Y	N	N	Y	N	N	Y	Y	Y
Peripheral neuropathy	Y	Y	NA	N	N	N	N	N	N	N	N	N	N
Facial dysmorphisms	N	N	N	N	N	N	Y	N	N	N	N	N	N
Spasticity	Y	N	Y	Y	Y	N	N	N	N	N	N	N	N
Respiratory distress	N	N	Y	N	Y	Y	N	N	N	N	N	N	N
Ocular manifestations	N	N	Cortical blindness	N	N	N	N	Strabismus	Nystagmus, loss of visual acuity	Loss of visual acuity	N	Nystagmus, strabism	Astigmatism
*Brain malformations*													
Basal ganglia abnormalities	Y	N	Y	Y	Y	Y	N	N	N	N	N	N	N
Cerebral brain atrophy	Y	N	Y	N	N	N	N	Y	Y	Y	N	N	Y
Cerebellar brain atrophy	Y	N	Y	N	N	N	N	N	N	N	N	N	N
*Therapy*													
Ketogenic diet	N	N	N	Y	Y	Y	N	Y	Y	Y	N	Y	N
Thiamine	N	Y	N	Y	Y	Y	Y	Y	Y	Y	Y	Y	Y
Arginine aspartate	N	Y	N	N	N	N	N	N	Y	Y	N	N	N
Antiepiletic drugs	N	N	Y	Y	Y	Y	Y	N	N	N	N	N	Y

DD/ MR/PMR—development delay, mental retardation, psychomotor retardation

Normal levels of plasma lactate—< 2.2 mmol/L

Normal levels of plasma pyruvate—< 0.180 mmol/L

Normal L/P ratio—< 20

Y—yes; N—no; NA—not available

^a^Twins

^b^Enzyme activity in fibroblasts

The 13 patients, seven males and six females, were born between 1983 and 2018, their current ages varying from 20 months to 36 years, with a median age of 15.5 years. Consanguinity was reported only in three families harboring *PDHX* mutations. Among this group of 13 patients, only two were siblings, born from a triplet pregnancy after in vitro fertilization (two affected males and one unaffected female) who carry a *PDHA1* mutation. No affected relatives are known in any of these families.

All patients showed the first symptoms either in the neonatal period (five individuals, 38.5%) or during infancy (eight individuals, 61.6%) with a median age of 0.5 years. In some cases however, the diagnosis was only achieved later, mainly among the older patients who presented a less severe clinical picture (five individuals confirmed between 3.3 and 17 years); indeed, the most striking example is the deficiency in E3 (also designated by dihydrolipoamide dehydrogenase, DLD), which was only diagnosed when the patient was 17 years old.

All patients but one are alive; the deceased patient is a boy carrying the c.1132C>T mutation in *PDHA1* (generating the p.R378C E1α variant); the death occurred at the age of 3.3 years and was caused by cardiorespiratory arrest.

### Genetic findings

In this cohort of 13 patients it was shown that patients carried mutations in three different genes: seven in the *PDHA1* gene, five in the *PDHX* gene, and one in the *DLD* gene (Table 1). The mutational spectrum revealed ten different mutations: five in *PDHA1*, three in *PDHX* and two in *DLD*.

As expected for an X-linked disorder, most patients with mutations in *PDHA1*, are males (6/7) in families with no consanguinity, but it is interesting to notice that in all these patients mutations arrived by a de novo mechanism, confirmed by parental sequencing, and that mosaicism was absent. On the other hand, most patients with mutations in *PDHX*, displaying an autosomal recessive mode of inheritance, are females (4/5) and consanguinity was detected in three cases. Finally, the single patient with mutations in *DLD*, also displaying an autosomal recessive mode of inheritance, is a compound heterozygous female with no consanguinity reported in her family.

In this patient cohort, all five different *PDHA1* mutations are missense (c.615C>G, c.757A>G, c.905G>A, c.1132C>T, and c.1133G>A), generating respectively the p.F205L, p.R253G, p.R302H, p.R378C and p.R378H E1α variants. The prevalence of missense mutations, in principle the less severe mutation type, strongly correlates with the majority of patients being males. On the contrary, the three mutations detected in *PDHX* gene (c.160+1G>A, c.483delC and c.850C>T), being nonsense (p.R284X) and frameshift (p.G26Vfs*7 and p.P161Pfs*17), usually result in absent protein, hence their highly deleterious consequences. Three out of the five patients are homozygous for the p.R284X variant, whereas one of the remaining patients is a compound heterozygote expressing the p.R284X and p.P161Pfs*17 variants, while the other is homozygous for the mutation generating the p.G26Vfs*7 variant. Regarding the two identified *DLD* mutations, both c.259C>T and c.803_804delAG are novel (Exome Variant, LOVD or ClinVar). The c.259C>T mutation generates the E3 p.P87S variant. Moreover, c.803_804delAG, originating a frameshift variant (p.Q268Rfs*3), is predicted to be very severe.

### Biochemical findings

All patients, regardless of the carried mutation, were suspected of PDC deficiency due to their elevated plasma lactate and pyruvate levels: lactate ranging from 3.0 to 17.0 mmol·L^−1^ (median value 6.6 ± 3.9) and pyruvate from 0.27 to 0.81 mmol·L^−1^ (median value 0.398 ± 0.086). Lactate/pyruvate ratios as expected, with a single exception, were ≤ 16, i.e., in the normal range. PDC deficiency was confirmed by determination of PDC activity. Enzymatic assays were performed either in circulating lymphocytes (12 patients) or cultured fibroblasts (two patients), both tissues having been analyzed in patient 11. All patients had their enzymatic activities confirmed in a second independent sample and the results always matched. Interestingly, a single false negative result was found in the lymphocytes of a female patient later identified with a *PDHA1* mutation. In our experience, the cut-off value for considering a primary PDC deficiency should be ≤ 30% of control activity and, indeed, all patients but one presented enzymatic activities ranging from 8.5% to 30% (median value 28.0 ± 7.9). The single exception was one of the siblings carrying the p.R378C variant in *PDHA1*, who presented 40% of normal control activity (Table 1), surprisingly the one whose symptoms manifested earliest in the neonatal period.

However, when stratifying the values of enzyme activity according to the affected PDC subunit, we observed some differences. Patients carrying *PDHX* mutations displayed higher and more similar values of relative activity (median value 29 ± 3.4%), whereas patients carrying *PDHA1* mutations displayed a wider range of activity (median value 23.5 ± 10.7%), with half of them below 20%.

### Clinical features

The clinical features observed in these patients’ cohort are displayed in Table 1 and, according to the most recently published data [6], can be roughly divided into two categories: one caused by *PDHA1* and *PDHX* mutations, and the other caused by *DLD* mutations. Indeed, the clinical phenotypes manifested by patients carrying *PDHA1* and *PDHX* mutations are quite variable and almost undistinguishable.

**Fig. 1:**
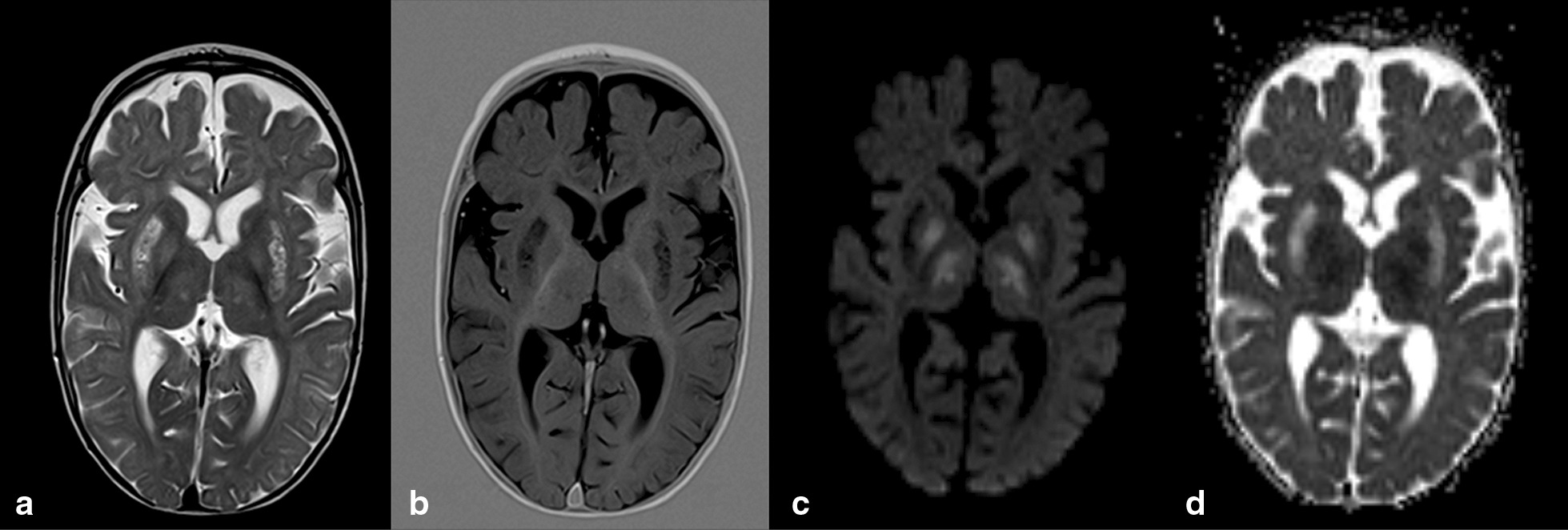
Magnetic resonance images of brain lesions of an 8 month old patient carrying a *PDHA1* mutation. Hyperintense bilateral and symmetrical lesions in the thalami, globus pallidus and putamina, on T2 weighted images, hypointense on T1 and with diffusion restriction suggestive of acute lesions; there is no diffusion restriction suggestive of chronic lesions. **a** axial T2, **b** axial T1 IR, **c** axial DWI, **d** axial ADC

**Fig. 2:**
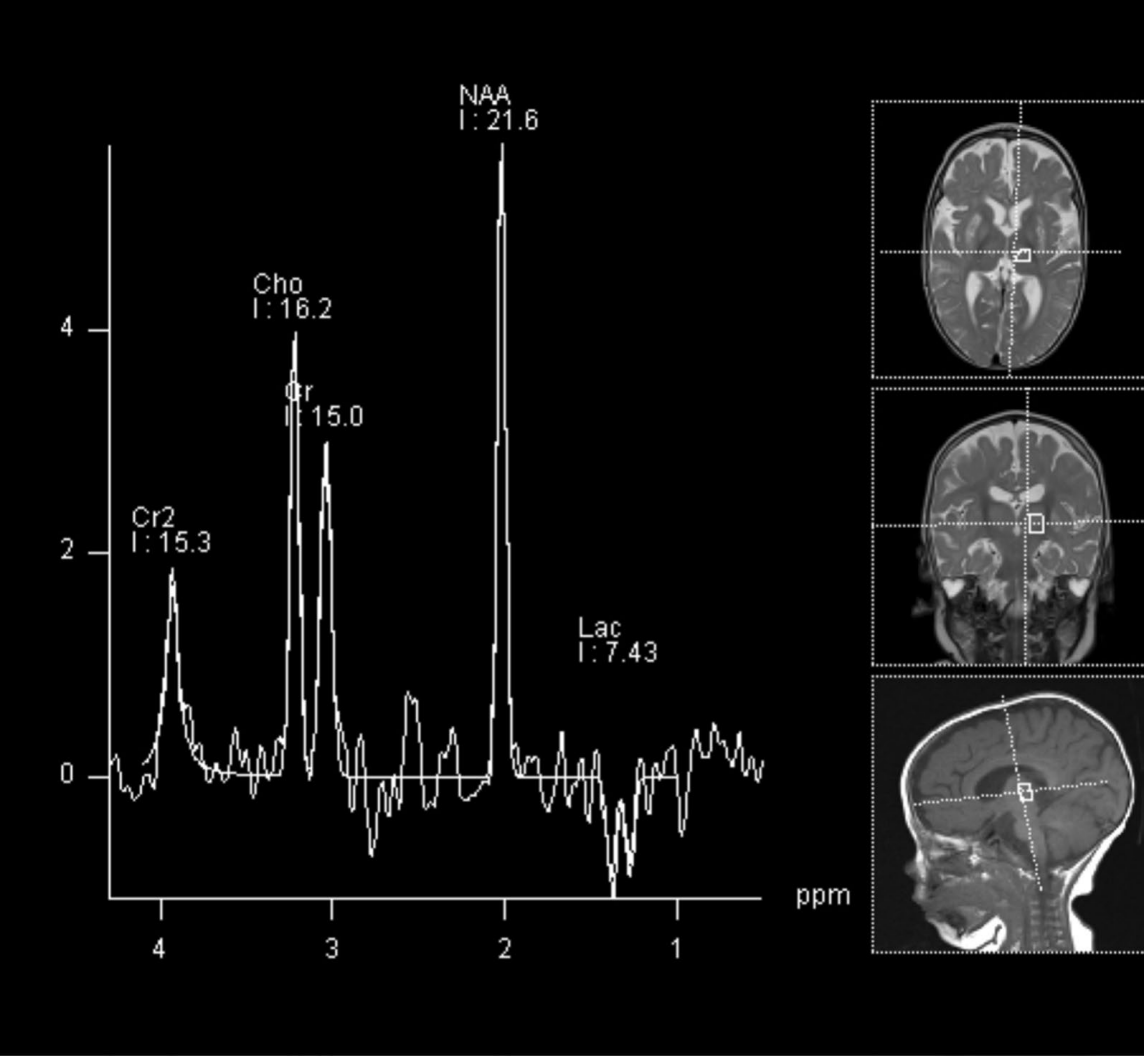
Magnetic resonance spectroscopy of a patient carrying a *PDHA1* mutation. Spectroscopy with TE = 135 ms in left thalamus; slight reduction of NAA, increased choline and lactate

Concerning neurological features, all individuals presented development delay/psychomotor retardation, which ranged from severe (eight patients: 61.5%) to moderate (four patients: 30.8%) or very mild (one patient who attended normal school: 7.7%). Hypotonia was also observed in all individuals, with a single exception (Patient 11) who nevertheless could display it at an earlier age. Seizures were reported in half the individuals, especially those carrying *PDHA1* mutations (5/7 patients: 71%). On the other hand, ocular manifestations were more frequent in individuals harboring *PDHX* mutations (4/5 patients: 80%). Microcephaly, dystonia and ataxia were also reported in half the patients of both these gene defects. As expected, facial dysmorphism was only detected in a single female patient carrying a *PDHA1* mutation. The patient with DLD deficiency revealed moderate developmental delay/psychomotor retardation, seizures, hypotonia, dystonia and ataxia. Basal ganglia abnormalities were exclusively observed in patients carrying mutations in *PDHA1*. Cerebral atrophy, but not cerebellar atrophy, was detected mainly in patients carrying *PDHX* mutations. However, in patients carrying *PDHA1* mutations, cerebral atrophy, when present, was always associated with cerebellar atrophy (Figs. 1 and 2). Patient 13, harboring *DLD* mutations, presented partial agenesis of *corpus callosum*.

### Treatment

All the patients are under therapeutic measures which include ketogenic diet, thiamine supplementation and antiepileptic drugs. Three patients with *PDHA1* mutations (3/7: 43%) and three with *PDHX* mutations (3/5: 60%) are under ketogenic diet with clearly beneficial effects on childhood-onset epilepsy or paroxysmal dystonia. The rationale for the ketogenic diet is that ketone bodies generated by fatty acid oxidation serve as an alternative energy substrate to glucose as the glycolytic end product, pyruvate, is not optimally metabolized. The long-term thiamine supplementation, as PDC cofactor, is prescribed in almost all patients (11/13), and antiepileptic drugs only in those presenting seizures (Table 1). Finally, it is interesting to mention that three of the patients carrying mutations in the *PDHA1* gene were under arginine aspartate supplementation, with beneficial effects at the physical and intellectual levels, especially Patient 2.

### In silico analysis of missense mutations

Bioinformatic analysis using the PolyPhen-2 server [19] suggested that all mutations but one affecting E1α subunit are most probably damaging. The E1α p.R302H, p.R378C and p.R378H variants displayed a score of 1.000 (sensitivity 0.00 and specificity 1.00), p.F205L displayed a score of 0.919 (sensitivity 0.81 and specificity 0.94), and p.R253G displayed a score of 0.007 (sensitivity 0.96 and specificity 0.75) thus being considered benign. As for E3 subunit, the p.P87S variant is predicted to be most probably damaging, with a score of 0.996 (sensitivity 0.55 and specificity 0.98).

**Fig. 3:**
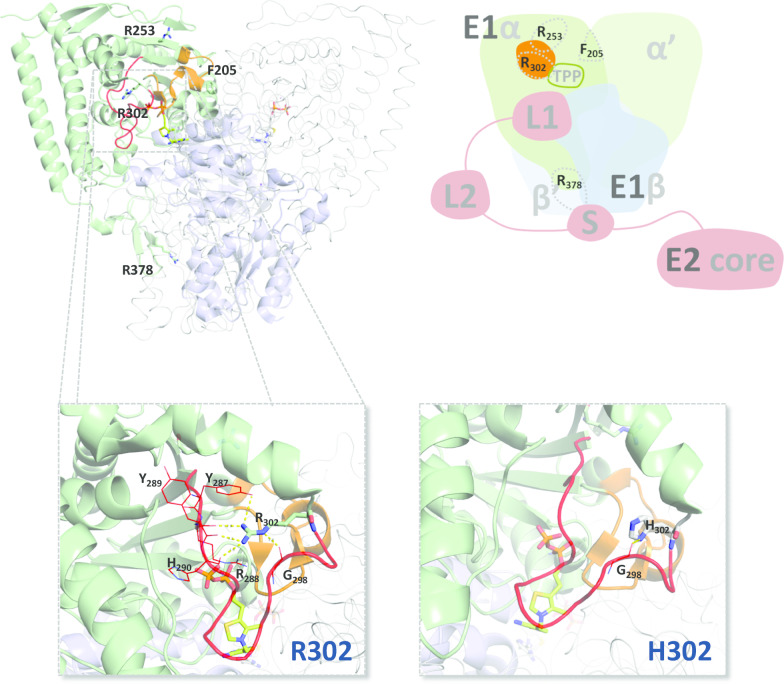
In silico analysis of pyruvate dehydrogenase complex E1 p.R302H variant. Top left panel, cartoon and ribbon representation of the heterotetrameric PDC E1 crystallographic structure (PDB entry 3EXE). E1α subunit represented in green; E1β represented in blue; E1α phosphorylation loop A represented in orange. The corresponding E1α′ and E1β′ subunits are represented as a gray ribbon. Residues that are substituted in variants identified in Portuguese PDC deficient patients with mutations in *PDHA1*, encoding the PDH E1α subunit, are represented in sticks. Top right panel, scheme representing the possible impact of substituted residues in pathogenic E1α variants: p.R253G substitution located near phosphorylation loop A (orange shape); p.F205L substitution possibly affecting αα′ interface (each E1α subunit represented in different shades of green); p.R302H substitution located in phosphorylation loop A close to the TPP cofactor binding site; p.R378C/H substitutions located close to the αβ interface (each E1β subunit represented in different shades of blue), and possibly affecting the interaction with a domain of the PDC E2 component. Bottom panel, zoom-in on the region surrounding R302 (left), showing its possible interactions with neighbouring residues (Y287, R288, Y289, H290 and G298), only the latter being retained upon substitution by H in the p.R302H variant (right). Structural model of PDC-E1 p.R302H variant was obtained by loading the structure of WT PDC-E1 (PDB entry 3EXE) into Pymol and applying the mutagenesis tool to generate all possible rotamers of the substituting amino acid side chain

To complement the information of the PolyPhen-2 server, which solely bases its predictions on the polypeptide sequence and overlooks other structural and functional details, such as e.g. cofactor binding and interaction with other proteins, we obtained and thoroughly inspected structural models of each E1α variant. The models obtained for the E1α p.R253G, p.R378C, p.R378H and p.F205L variants have been recently described by our group, attempting to understand the molecular mechanisms underlying the pathogenicity of the corresponding mutations [20]. All mutations result in putative loss of H-bonds, electrostatic or hydrophobic interactions between the side chains of the substituted amino acids and neighboring residues, with predicted effects on P-loop destabilization, inter-subunit interactions and proper oligomeric assembly, as well as interaction with other PDC components. Herein, we additionally generated a structural model of the E1α p.R302H variant (Fig. 3). R302 is located at one end of the P-loop A, its side chain being within electrostatic and H-bonding distance to the side chain or main chain carbonyl moieties of Y287, R288, Y289, H290 (active site residue) and G298, all residues belonging to the same P-loop. Upon substitution by a histidine residue, most of the possible interactions between its side chain and other residues in the P-loop are lost. The single remaining H bond is that between the side chain imidazole and the main chain carbonyl of G298. Therefore, in the p.R302H E1α variant, the net loss of four possible side chain interactions with other P-loop A residues (Fig. 3) is likely to contribute to a more disordered loop and consequently lower enzymatic activity. Notably, the degree of disorder in P-loop A has been negatively correlated with E1 enzymatic activity, since an ordered loop favors TPP binding, which itself promotes P-loop A order [21].

**Fig. 4:**
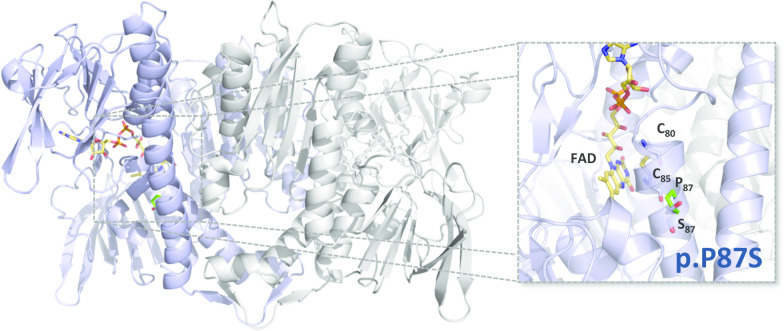
In silico analysis of pyruvate dehydrogenase complex E3 p.P87S variant. Cartoon representation of homodimeric PDC E3 crystallographic structure (PDB entry 6I4T; one monomer represented in blue, the other in grey). Flavin adenine dinucleotide (FAD) cofactor in yellow sticks. Right panel, zoom-in on the location of the P87S substitution. P87 is located in an α-helix which contains the active site cysteine residues C80 and C85. Substitution of P by S will likely affect the helix structure and disturb the proximity between the active site disulphide and the FAD cofactor. Structural model of PDC-E1 p.R302H variant was obtained by loading the structure of WT PDC-E3 (PDB entry 6IT4) into Pymol and applying the mutagenesis tool to generate all possible rotamers of the substituting amino acid side chain

To further understand the pathogenicity of the *DLD* mutation generating the E3 p.P87S variant, a structural model was obtained (Fig. 4) based on the reported 3D crystallographic structure of another disease-causing E3 variant (PDB entry 6I4T) [22]. As observed in Fig. 4, P87 is located in a helix that lines with the flavin adenine dinucleotide isoalloxazine ring and contains the active site disulfide composed of C45 and C50. Substitution of P87 by a serine is likely to alter the flexibility and thus the overall stability of the respective helix, possibly affecting the enzymatic activity.

## Discussion

The diagnosis of PDC deficiency is extremely challenging due to a phenotypic presentation that can be observed in many other neurological disorders, especially those causing abnormal mitochondrial metabolism [6, 23, 24]. Indeed, PDC deficiency can be included in the vaster group of pyruvate oxidation defects (POD) which also involves defects in genes coding for proteins participating in the whole pyruvate oxidation route, including cofactors, regulation of PDC and the mitochondrial pyruvate carrier [6]. Nevertheless, in this study, we only included patients carrying mutations in the genes encoding the PDC subunits (*PDHA1*, *PDHB*, *PDHX*, *DLAT* and *DLD*). Accordingly, and comparing the prevalence rate we obtained *versus* the previously reported prevalence (respectively 1:790,000 and 1:1,000,000) we may assume that no significant number of patients were missed.

In such context, we aimed to present the first report on a cohort of PDC deficient Portuguese patients combining information on the associated clinical, biochemical, enzymatic and genotypic spectra. The mutational spectrum of PDC deficiency in this group of patients revealed ten different mutations affecting three genes, *PDHA1* (five), *PDHX* (three) and *DLD* (two). Concerning the prevalence of the deficiency of each gene, these data generally agree with literature surveys [6, 9] and also with several studies focused on different populations [8, 25, 26]. However, the frequency of mutations in the *PDHA1* gene is lower than the usually reported average of 75–80%, due to a high number of patients harboring mutations in the *PDHX* gene (38% in our cohort versus 10% in the literature).

The most striking evidence is the relatively high incidence of E3BP deficiency, mostly caused by a p.R284X E3BP variant, half the cases originating from the Azores Islands, thus denoting a founder effect. This E3BP variant was first described by our group [27] and, until now, only another Portuguese patient has been reported to carry this mutation [28]. Furthermore, the mutational spectrum of E3BP deficiency in Portugal includes very severe mutations, leading to null alleles. Nevertheless, our older patients surprisingly reached adulthood, in line with the high proportion of long-term survival among reported E3BP deficient patients [8, 29, 30]. In general, an overwhelming majority of the mutations hitherto identified in the *PDHX* gene are, as in this work, deletions, nonsense mutations, point mutations at intron–exon boundaries, or even large intra-genic rearrangements, expected to result in a complete absence of E3BP protein [8, 30–32]. Despite this fact, the patients retain considerably significant PDC activity (20–30%), considering the expected impairment on PDC assembly. On the one hand, as a structural subunit devoid of enzymatic activity, E3BP does not directly contribute to the complex catalytic activity. On the other hand, the significantly truncated E3BP, if present at all in the cell, would likely compromise the structure of the E2/E3BP PDC core and binding of the E3 component. Both E2 and E3BP components have a similar structure and domain organization, despite only E2 being catalytically active [33]. However, the possibility of the E2 core directly binding to the E3 enzyme may underlie the observed residual PDC activity [30–32, 34]. In their recent work, Prajapati and collaborators report a non-uniform stoichiometry of the E2/E3BP PDC core. The imbalanced distribution of E2 and E3BP constituents of the trimeric units results into structurally dynamic E1 and E3 clusters [35]. Moreover, for one of the proposed models of *E. coli*, the PDC core is a fully functional E2 homotrimer operating in a “division-of-labor” mechanism, including binding of the E3 component [35, 36].

Concerning the mutational spectrum of E1α deficiency, five different *PDHA1* mutations were identified, all but a single one (c.1132C>T, encoding the p.R378C variant) from non-consanguineous patients. Almost all mutations affect an arginine codon [37] and those located in exons 10 and 11 cause a severe phenotype, because the resulting protein variants present very low enzyme activity. On the contrary, the two mutations located in exon 7 originate moderate (c.615C>G, encoding the p.F205L variant) or very mild (c.757A>G, encoding the p.R253G variant) phenotypes. Interestingly, mutations affecting codon 378 are considered particularly lethal [9]. Indeed, from our male patients carrying the p.R378C mutation, one deceased at three years of age and the twins, presently aged 8 years, display a severe clinical picture. However, a female patient bearing the p.R378H substitution reached the adulthood, probably due to a lyonization effect.

Regarding a possible genotype-dependent phenotypic presentation, our data is roughly suggestive of such a correlation. Effectively, the patients harboring the most deleterious mutations (c.905G>A and c.1132C>T in *PDHA1* and all the mutations in *PDHX*) present the most severe phenotypes, involving serious psychomotor retardation, hypotonia and seizures, whereas those carrying less severe mutations accordingly display a better clinical outcome. The most puzzling observation concerns the female carrying mutations in *DLD*. Despite being a compound heterozygote bearing two severe mutations, her clinical course was reasonable until 2018 when she suffered an acute metabolic decompensation originating spastic tetraparesis with gait and language loss. Although she partially recovered language, she currently presents a moderate-to-severe psychomotor handicap. Despite the E3 subunit being common to other enzyme complexes such as α-ketoglutarate dehydrogenase and branched-chain amino acid dehydrogenase, this patient did not display the associated biochemical or clinical phenotypes.

Irrespectively of our patients’ cohort size, the majority of our PDC deficient patients remarkably reached adulthood, as opposed to several other reports [9, 18, 25, 26]. Concerning the therapeutic measures to which these individuals are subjected, it is clear they are only palliative, since all patients but one continue presenting clinical features ranging from moderate to severe forms. The single exception is Patient 2 who seems to represent an exceptional case, because his treatment only encompasses thiamine (E1 subunit cofactor) and arginine aspartate supplementation [38]. Arginine aspartate (Asparten®, Sargenor®) is an anti-asthenic over-the-counter medicine. Aspartate is considered an anaplerotic agent, whereas arginine has the ability to suppress aggregation during protein folding by binding to the folding intermediates through weak interactions, thus being considered a putative chemical chaperone [39–41]. This patient carries the *PDHA1* c.757A>G mutation that originates the p.R253G E1α variant, whose in silico and in vitro analyses with the recombinant protein exhibited lower affinity for TPP and lower residual enzymatic activity, in addition to increased proneness to aggregation, in comparison with WT PDC-E1 [20]. The same impairment on the affinity for TPP was observed in various *PDHA1* missense mutations [20]. Thiamine supplementation is thus likely to ameliorate the functional impact in terms of TPP affinity for several of these variants [20]. Nevertheless, our observation of the serious regress of the clinical symptoms, while the arginine aspartate uptake was interrupted in Patient 2 [38], suggests that this patient benefits from both arginine aspartate and thiamine treatments.

In conclusion, the identification of the disease-causing mutations, together with the functional and structural characterization of the respective protein variants, allows getting insight on the severity of the clinical phenotype and the selection of the most appropriate therapy, namely the option for a ketogenic diet.

## Materials and methods

### Cohort of patients

This study included all PDC deficient patients whose diagnosis was confirmed at the molecular level: thirteen individuals comprising a pair of monozygous twin siblings, 6 being females and 7 males. Since patients originated from all regions of Portugal, this cohort can be considered representative of the whole population. The diagnosis of patients, suspected due to high lactate and pyruvate plasma levels and respective lactate/pyruvate ratio < 20, was confirmed by reduced PDC activity (*ca* < 30% of laboratory control mean: 1734 ± 455 (range: 1279 – 2189) pmol·min^−1^·mg protein^−1^; n = 70) in peripheral lymphocytes and/or cultured fibroblasts originating from skin biopsy, and also by identification of the causative mutation(s).

This study was approved by the local Ethics Committees and informed consents were obtained from the patients or their parents, who were also enrolled in the study, whenever necessary and possible. Declaration of Helsinki was also strictly observed.

### Phenotypic evaluation

The physicians following these patients completed a questionnaire involving a wide range of parameters, namely: general characteristics of the patients, clinical features, brain malformations, biochemical findings, genetic findings, and current therapy.

### Sample collection and preparation

Blood samples were collected after overnight fasting by venipuncture into EDTA- and perchloric acid-containing tubes for plasma separation and quantification of lactate and pyruvate levels, respectively, and into heparin-containing tubes for peripheral blood mononuclear cells (PBMC) isolation.

PBMC were separated at room temperature on a Ficoll-Paque gradient. Fibroblasts were cultured in Dulbecco's Modified Eagle Medium supplemented with 10% newborn calf serum and 1% antibiotic/antimycotic solution. Pelleted cells were resuspended in homogenization buffer (80 mM KH_2_PO_4_, pH 7.4, 2 mM EDTA). PBMC suspensions were immediately disrupted by sonication, whereas fibroblast suspensions were treated with 5 mM dichloroacetate for 15 min at 37 °C. The reaction was blocked by addition of a stopping solution (25 mM NaF, 25 mM EDTA, 4 mM DTT), and cells were disrupted by three freeze/thaw cycles.

### PDC activity assay

Enzymatic activity was measured using a radiochemical method based on the release of ^14^CO_2_ from [1-^14^C]-pyruvate [42] with minor modifications (Johannes Mayr and Wolfgang Sperl, personal communication). Briefly, 100 µL of cell homogenates were incubated at 37 °C for 10 min in 100 µL of reaction buffer (32 mM phosphate buffer containing 4 mM MgCl_2_, 2 mM CaCl_2_, 0.5 mM NAD^+^, 0.5 mM TPP, 0.1 mM CoA and 5 mM carnitine; final concentrations); blanks were obtained by replacing the cell homogenate with homogenizing buffer. Then, the reaction was started by addition of 50 µL [1-^14^C]-pyruvate solution (0.5 mM, 0.067 µCi) and allowed to proceed for 30 min, after which the reaction was stopped by addition of 80 µL 6 N H_2_SO_4_. The released ^14^CO_2_ was trapped in filter paper saturated with benzethonium hydroxide, for 15 min post-incubation at room temperature and under gentle stirring, and its amount measured in a scintillation counter. All samples were analyzed in triplicates and PDC activity was expressed in pmol·min^−1^·mg protein^−1^.

### Preparation of genomic DNA, RNA and cDNA

Genomic DNA and, eventually, total RNA were isolated from peripheral blood leukocytes using the Puregene Cell and Tissue kit (Gentra Systems) and the Trizol method, respectively; 5 μg of total RNA were used for the reverse transcription reaction (Amersham First Strand cDNA Synthesis kit, GE Healthcare Bio-Science Corp.).

### PCR amplification of PDC coding genes

The complete sequence of each gene was obtained by PCR amplification of individual exons, including intronic boundaries, or overlapping fragments of the respective cDNA. The PDC subunits under analysis together with their coding genes, approved symbols and reference sequences are listed in Additional file 1: Table S1, whereas primer sequences designed for each gene amplification are listed in Additional file 1: Table S2.

### Sequence analysis

PCR and RT-PCR products were purified from solution or directly from agarose gels, using Isolate II PCR and Gel Kit (Bioline). PCR forward or reverse primers were added to the purified products from each individual sample and submitted to bi-directional Sanger sequencing. All chromatograms corresponding to PCR and RT-PCR fragments were analyzed with BLAST (NCBI).

### In silico analysis of PDC-E1 mutations

To better establish genotype–phenotype correlations regarding the missense mutations in *PDHA1*, we undertook an in silico analysis of protein variants resulting from the described mutations. Besides evaluating the potential pathogenicity of the mutations using the PolyPhen-2 server [19], we sought to obtain structural models of the protein variants through two complementary strategies, previously described for the p.R253G, p.R378C, p.R378H, and p.F205L variants [20], and herein extended to p.R302H: i) submitting the sequence of the amino acid substituted variant to the SwissModel server and retrieving the corresponding model; and ii) using the Mutagenesis tool in Pymol (version 1.7) [43] employing the structure of WT PDC-E1 (PDB entry 3EXE) as template [21].

## Supplementary information

**Additional file 1:** List of the analyzed PDC components showing the type of enzymatic activity, the Enzyme Commission (EC) number, the gene symbol, the HUGO identification, the reference sequences and the chromosomal localization (Table S1) and list of primers used in this study (Table S2).

## Data Availability

Data can be made available upon reasonable request to the corresponding authors.
