# Supplementary material for: Pyruvate dehydrogenase complex deficiency: updating the clinical, metabolic and mutational landscapes in a cohort of Portuguese patients
Source: Orphanet J Rare Dis. 2020 Oct 22;15:298. doi: 10.1186/s13023-020-01586-3 (PMC7579914; doi:10.1186/s13023-020-01586-3)
Supplement: Supplementary file 1 — Additional file 1: List of the analyzed PDC components showing the type of enzymatic activity, the Enzyme Commission (EC) number, the gene symbol, the HUGO identification, the reference sequences and the chromosomal localization (Table S1) and list of primers used in this study (Table S2). [file 13023_2020_1586_MOESM1_ESM.docx]

**Table S1.** List of the analyzed PDC components showing the type of enzymatic activity, the Enzyme Commission (EC) number, the gene symbol, the HUGO identification, the reference sequences and the chromosomal localization.

| **Component / subunit** | **Enzymatic Activity** | **Enzyme Commission number** | **Approved Gene Symbol** | **HGNC ID** | **GenBank** | | **Chromosomal Location** |
| --- | --- | --- | --- | --- | --- | --- | --- |
| **E1α** | pyruvate dehydrogenase (acetyl-transferring) | EC 1.2.4.1 | *PDHA1* | 8806 | NM_000284.4 | NG_016781.1 | Xp22.12 |
| **E1β** |  |  | *PDHB* | 8808 | NM_000925.4 | NG_016860.1 | 3p14.3 |
| **E2** | dihydrolipoyllysine-residue acetyltransferase | EC 2.3.1.12 | *DLAT* | 2896 | NM_001372031.4 | NG_013342.1 | 11q23.1 |
| **E3** | dihydrolipoyl dehydrogenase | EC 1.8.1.4 | *DLD* | 2898 | NM_000108.5 | NG_008145.1 | 7q31.1 |
| **E3BP** | - |  | *PDHX* | 21350 | NM_001135024.1 | NG_013368.1 | 11p13 |

**Table S2.** List of primers used in this study.

**_____________________________________________________________________**

**cDNA amplification**

***PDHA1* messenger** (2 fragments)

PDHA1-5’-F 5’ – GGGCACCTGAAGGAGACTT – 3’

PDHA1-R 5’ – CTTTAGTTCTTCCACACTGG – 3’

PDHA1-F 5’ – AGTGGATGGAATGGATATCC – 3’

PDHA1-3’-R 5’ – GTCTGGTAGCCCCCTGAAGG – 3’

***PDHX* messenger** (2 fragments)

PXF2-F 5’ – CTGCTGCGTTATCTTGTGGGCT – 3’

PXW2-R 5’ – TGAGTGAATGTGCCCACTGCATTG – 3’

PXP2-F 5’ – CAATGCAGTGGGCACATTCACTGA – 3’

PXR2-R 5’ – TAACAACTACTGAATCAACTAAGC – 3’

***DLD* messenger** (2 fragments)

E3A-F 5’ – AGCGGAGAAAGTATTGGCGGA – 3’

E3D-R 5’ – TTTAGTTTGAAATCTGGTATTGAC – 3’

E3D-F 5’ – CAGCAGTTGAACGTTTAGGTCATG – 3’

E3F-R 5’ – TCTTGGAGCTGTGAGAATATCCT – 3’

______________________________________________________________________

**Genomic DNA amplification**

***PDHA1* gene**

PDHA1-1-F 5’ – GCGCAGCGCATGACGTTATTACG – 3’

PDHA1-1-R 5’ – CCGGCCAGCCCGGGAGGTCT – 3’

PDHA1-2-F 5’ – GCCAAAGCATGGATTCATTT – 3’

PDHA1-2-R 5’ – TCTGAACTTCTGATCCTGGACA – 3’

PDHA1-3-F 5’ – CCAAGCCCCATCTCATTG – 3’

PDHA1-3-R 5’ – ACACAGTTCCACCACAAACC – 3’

PDHA1-4-F 5’ – TTATTGCTTCTGGTTTGGGC – 3’

PDHA1-4-R 5’ – CCCCTTTCTGTAAATCAACAGC – 3’

PDHA1-5-F 5’ – TGGTTGAGCCTCAGAGTACA – 3’

PDHA1-5-R 5’ – TGGCTGTACTAGCTTCAGGA – 3’

PDHA1-6-F 5’ – GATTCTGGCCAGGAGTGAAA – 3’

PDHA1-6-R 5’ – GGTGAGCTCCTTCACAGGAA – 3’

PDHA1-7-F 5’ – AGGAGGCCTTTCTGTGCTTT – 3’

PDHA1-7-R 5’ – CGGCCCCACCACAGGGTTCCT – 3’

PDHA1-8-F 5’ – TGTCGCCCCTCCCCTGTTTAT – 3’

PDHA1-8-R 5’ – CTTCCATCTCATGCACCTCA – 3’

PDHA1-9-F 5’ – TGAGCCACCATCCTGGCCTT – 3’

PDHA1-9-R 5’ – GCGTACATGAAGTGAACTGG – 3’

PDHA1-10-F 5’ – ATTTCACTCATTGGGACATCC – 3’

PDHA1-10-R 5’ – TGGTTCACAGTCCACCAAAA – 3’

PDHA1-11-F 5’ – TTTTGGTGGACTGTGAACCA – 3’

PDHA1-11-R 5’ – GTCTGGTAGCCCCCTGAAGG – 3’

***PDHX* gene**

PX1F 5’-AGAGACCTAAAGGCACCGCT-3’

PX1R 5’-AAGCAGGCCCTCAATCATAA-3’

PX2F 5'-TGGGAATCTTTTAGACTTTGGA-3’

PX2R 5’-TGCTGAACCCAGAAAACCTT-3´

PX3F 5’-CAACCCAGAAATAGCTACGGA-3’

PX3R 5’-CACATTAAAAATAAGGAGGCAAAA-3’

PX4F 5’-TGCAGTCATGGGGTTTTACTT-3’

PX4R 5’-ACAGCAACTTCCTACGTGATG-3’

PX5F 5’-GTGACCATCTGTGGGAGTCA-3’

PX5R 5’-TTATTCAGAAAACAACTCTTGCAT-3’

PX6F 5’-TCACCTGCGTTTTCTGAAAGT-3’

PX6R 5’-GTGAGCCAAGATTGTGCCAT-3’

PX7F 5’-TTCCACTTGTGGTTTAACGGA-3’

PX7R 5’-TTTCCTCTAGCACAAATATACCCA-3’

PX8F 5'-ACAAGTTTGAAGTTGTAATGGTCA-3’

PX8R 5’-GAGGGAGATCAAACGATAGGA-3´

PX9F 5’-TTTTTCTGTAACCGCCTTGG-3’

PX9R 5’-TCTCCCCTTCACACACACAA-3’

PX10F 5’-GGTAACAAAATCAAATCAAGGCA-3’

PX10R 5’-TTCAGATAAATGAAAGGCTGACA-3’

PX11F 5’-ACGGAAAGGGGACTTTGATT-3’

PX11R 5’-TTGAGGACTAGGCAAGTCGG-3’

***DLD* gene**

DLD1-F 5’ – CTCCCGGGTGATGACGTA – 3’

DLD1-R 5’ – CTCCCGGGTGATGACGTA – 3’

DLD2-F 5' – TTGATACGTTTGCCCAAAAT – 3’

DLD2-R 5’ – ATTGAAATAGAAAGGAACTGTCAG– 3´

DLD3-F 5’ – TGCTGATTTGTACTGTAAGAGGTT – 3’

DLD3-R 5’ – TGATCAACCCTTCCCAAATTA – 3’

DLD4-F 5’ – CCGAATAGCTTGTTTTGTAGAAG – 3’

DLD4-R 5’ – TTGTCTAATCTAGTCTCAATCCAT – 3’

DLD5-F 5’ – GAACGAAACTCCGTCTCAAAA – 3’

DLD5-R 5’ – TCTTTAGACAGAAGAGCCAAGTCA – 3’

DLD6-F 5’ – TTGGTGAGTGAAAAACACTGC – 3’

DLD6-R 5’ – TCCCCTGTAACCAAGTTCAAA – 3’

DLD7-F 5’ – AAGTAAGGAAGCATTTTGTTTTAG – 3’

DLD7-R 5’ – TCAGTCAGAAATCTCTCAAAGTTC – 3’

DLD8-F 5' – TCTGCAAATTTGGAACCCAT – 3’

DLD8-R 5’ – GGACCTTTAAGTCCCTTCCAA– 3´

DLD9-F 5’ – AAGATGATTTCGTAAACATTTGCT – 3’

DLD9-R 5’ – TTGCTTAAAGAGACAGGGATGA – 3’

DLD10-F 5’ – CTTGAGAAATTGCTGGCCTT – 3’

DLD10-R 5’ – TTCCCCAAAGCCAATACATA – 3’

DLD11-F 5’ – TTTTTGGTGACTTGTTTACTGGAA – 3’

DLD11-R 5’ – TGCTGTTTCTCAACCACCAA – 3’

DLD12-F 5’ –TCCTTCTATGTGCTTTGCGA– 3’

DLD12-R 5’ – TCTTAATGGGATTTCTCTGGTTTT – 3’

DLD13+14-F 5’ – CCCCTCAACAATTGCTATCC – 3’

DLD13+14-R 5’ – TGGAGCTGTGAGAATATCCTGTT – 3’

______________________________________________________________________
